# NOTCH1 and UPR signaling in embryonic heart development under maternal high-fat diet influence

**DOI:** 10.3389/fmed.2025.1620495

**Published:** 2025-12-04

**Authors:** Yupeng Shi, Bingyu Li, Qingyun Shi

**Affiliations:** 1 Department of Obstetrics, Beijing Obstetrics and Gynecology Hospital, Capital Medical University, Beijing, China; 2 Beijing Maternal and Child Health Care Hospital, Beijing, China; 3 Beijing Obstetrics and Gynecology Hospital, Capital Medical University, Beijing, China

**Keywords:** maternal high-fat diet (HFD), developmental origins of health and disease, NOTCH1, unfolded protein response (UPR), inositol-requiring protein 1α (IRE1α)

## Abstract

**Introduction:**

During the submission and review period, I obtained my Master’s degree from Beijing Obstetrics and Gynecology Hospital, Capital Medical University, and subsequently joined the Obstetrics Department of the same hospital. Due to this change in status from student to hospital staff, I am now required to use my staff email for correspondence, which has been updated in the manuscript. In addition, our hospital has revised the official format of the institution’s name for submissions, and accordingly, the affiliation in the manuscript has been updated from “Beijing Obstetrics and Gynecology Hospital, Capital Medical University” to “Beijing Obstetrics and Gynecology Hospital, Capital Medical University. Beijing Maternal and Child Health Care Hospital.” We apologize for any inconvenience these changes may have caused to the editorial team.

**Objective:**

This study aimed to investigate how maternal high-fat diet (HFD) during pregnancy affects embryonic heart development.

**Methods:**

C57BL/6J female mice were fed a HFD before and during pregnancy. Maternal blood was collected at P5.5, P10.5, and P14.5 to assess lipid levels. Embryonic hearts at E14.5 were examined by H&E staining, and ventricular protein expression of NOTCH1 and UPR-related molecules was measured via Western blot. E14.5 cardiomyocytes were cultured to evaluate NOTCH1 expression after IRE1α pathway inhibition.

**Results:**

Compared with the control group (Group A), serum levels of TC, TG, and LDL-C were increased, and HDL-C was decreased in maternal mice fed a high-fat diet during pregnancy (Group B) and a high-fat diet both before and during pregnancy (Group C). Group B embryos exhibited abnormal ventricular wall compaction, thinning, and valve defects, which were more severe in Group C. NOTCH1 expression was reduced in B and C ventricular tissues, while XBP1s and apoptosis-related proteins caspase-3/7 were elevated. Inhibition of the IRE1α pathway abolished differences in NOTCH1 expression among groups in cultured cardiomyocytes.

**Conclusion:**

Maternal HFD before and during pregnancy induces abnormal embryonic heart development, likely via IRE1α pathway activation in the UPR, which suppresses NOTCH1 expression and promotes apoptosis. These findings underscore the importance of a balanced maternal diet for proper embryonic heart development.

## Introduction

Congenital heart disease (CHD) is one of the most prevalent malformations among newborns and a leading cause of mortality due to congenital anomalies. From 1990 to 2021, the mortality rate among CHD patients has significantly decreased, primarily due to advancements in the diagnosis and management of CHD, rather than a reduction in its incidence. The incidence of CHD has remained stable over the long term (1). In addition to genetic factors, environmental influences also contribute to the occurrence of CHD. For example, excessive maternal alcohol consumption during pregnancy, diabetes (2), and obesity are all factors that can increase the prevalence of CHD (3). Maternal obesity represents a significant global public health issue, associated with various adverse pregnancy outcomes (4). A growing body of research indicates that maternal obesity can adversely affect fetal heart development, with the incidence of congenital heart disease rising in parallel with rates of maternal obesity (5, 6). These sharp increases can be partly attributed to dietary patterns associated with obesity. It has been clearly demonstrated that excessive maternal nutrition during pregnancy leads to an increased incidence of CHD in offspring, and a high-fat diet in mothers can elevate the risk of heart defects in their descendants (7). However, the precise molecular mechanisms underlying these effects remain unclear.

According to the Developmental Origins of Health and Disease (DOHaD) theory, the origins of lifestyle-related diseases are established during the stages of fertilization, embryonic development, fetal growth, and the newborn period through the interaction between genetic factors and environmental influences, such as nutrition, stress, and environmental chemicals (8). During the fetal period, tissues and organs undergo rapid development and growth. The heart, one of the earliest organs to form during development (9), undergoes complex signaling processes that require precise temporal and spatial expression of relevant signaling molecules. Consequently, exposure to adverse maternal environments during critical windows of offspring development can disrupt normal heart development and increase the risk of heart disease in offspring. Research indicates that a high-fat diet (HFD) consumed by mothers during pregnancy can trigger inflammatory responses in their offspring (10), leading to lipid metabolism disorders. Animal studies have demonstrated that when pregnant female mice are fed a HFD, fat accumulation occurs in the myocardial cells of their offspring, accompanied by an increased expression of the apoptosis marker protein caspase-3 (11). Furthermore, lipid accumulation can induce endoplasmic reticulum (ER) stress (12).

The ER, which serves as the site for lipid synthesis, protein folding, and assembly, faces continuous challenges from physiological demands and pathological damage. Abnormal lipid accumulation within cells can disrupt ER function, leading to a state of stress (13). Endoplasmic reticulum stress represents a protective response aimed at restoring protein homeostasis through the activation of the unfolded protein response (UPR). Initially, heat shock proteins, such as GRP78 and GRP94, are recruited to assist in the folding of nascent proteins. Subsequently, the three primary sensors of ER stress–inositol-requiring protein 1α (IRE1α), protein kinase RNA-like ER kinase (PERK), and activating transcription factor 6 (ATF6)–are activated. These sensors further activate additional proteins, including spliced X box-binding protein 1 (XBP1s) and phosphorylation of eukaryotic translation initiator factor 2α (phospho-EIF2α), to facilitate molecular adaptation to stress conditions. This process is referred to as the UPR (14). The UPR alleviates the burden of unfolded or misfolded proteins and restores protein homeostasis. However, if ER stress persists, the prolonged activation of the UPR can induce programmed cell death, or apoptosis, to protect the organism by eliminating stressed cells (15).

In this study, we present evidence that a high-fat diet consumed by mothers before and during pregnancy leads to abnormal embryonic heart development. This is primarily characterized by inadequate ventricular wall compression, thinning of the ventricular walls, and maldevelopment of the heart valves. Such abnormalities are likely attributable to the adverse intrauterine environment induced by the high-fat diet, which activates the IRE1α pathway of the UPR in the embryonic heart. This activation results in reduced expression of NOTHC1, thereby adversely affecting normal heart development. Our findings highlight the crucial role of maintaining a well-balanced dietary pattern before and during pregnancy, which may serve as a potential strategy to lower the risk of CHD.

## Materials and methods

### Animal experiment

This study received approval from the Ethics Committee of Beijing Obstetrics and Gynecology Hospital, Capital Medical University. All animal experiments were sanctioned by the Animal Ethics Committee of the same institution (Approval No.: BOGH21-2407-1), and measures were implemented to minimize both the use of and suffering experienced by the animals. C57BL/6 mice were sourced from Beijing Vital River Laboratory Animal Technology Co., Ltd. They were maintained in a specific pathogen-free clean animal facility at a temperature range of 22°C–25°C, with constant access to food and water. Mice were fed either a control diet (D12450B) or a HFD (D12492) obtained from Beijing Keao Xieli Feed Co., Ltd. The control diet provided 20% of energy from protein, 70% from carbohydrates, and 10% from fat, whereas the HFD provided 20% of energy from protein, 20% from carbohydrates, and 60% from fat (detailed ingredient composition is shown in Supplementary Table 1).

Female C57BL/6 mice were randomly assigned to three groups: the A group, the B group, and the C group, each consisting of six female mice. The feeding regimen for the A group involved feeding the mice a control diet for 8 weeks prior to pregnancy, which continued throughout the gestation period. In contrast, the B group received a control diet for 8 weeks before pregnancy, followed by a switch to a HFD during gestation. The C group was subjected to a HFD for 8 weeks prior to pregnancy, which was maintained throughout the pregnancy. All male mice were fed a control diet. After 8 weeks on their respective diets, female mice from each study group were mated with age-matched male mice that had been fed a control diet. Pregnancy was confirmed by the presence of sperm in vaginal smears the following morning, and this day was designated as pregnant day 0.5 (P0.5), also referred to as embryonic day 0.5 (E0.5).

### Serological test

Blood samples were collected from the tail tips of maternal mice at P5.5, P10.5, and P14.5. The samples were centrifuged at 3000 rpm for 15 min, and the supernatant was obtained for measuring the levels of total cholesterol (TC), triglyceride (TG), low-density lipoprotein cholesterol (LDL-C), and high-density lipoprotein cholesterol (HDL-C) in the serum. All kits used for these analyses were procured from Shenzhen Mindray Bio-Medical Electronics Co., Ltd.

### Cardiac histology and immunofluorescence analysis

Pregnant mice at P14.5 were euthanized using carbon dioxide, and the embryos were subsequently collected. Following a washing step, the embryos were fixed in 4% neutral formaldehyde for 24 h, embedded in paraffin, and sectioned into 4 μm slices, which were then sequentially mounted on slides. The paraffin sections were deparaffinized, followed by H&E staining, dehydration through a graded series of ethanol, clearing in xylene, and mounting with neutral resin. The images were observed using an inverted microscope (Ci-S, Nikon) and quantitatively analyzed using ImageJ software. Immunofluorescence is used to identify Notch1. Subsequently, the slices were incubated with the corresponding fluorescent dye-labeled secondary antibody for 2 h. Nuclei were visualized using DAPI and fluorescence was visualized using a Zeiss AxioImager II microscope.

### Protein extraction and western blot analysis

The tissue was washed twice with PBS, cut into small pieces, and lysed on ice using RIPA buffer (150 mM NaCl, 1% Triton X-100, 0.1% SDS, 0.5% sodium deoxycholate, 50 mM Tris-HCl, pH 8.0), supplemented with Xpert Protease Inhibitor Cocktail Solution (100X) (GenDEPOT) for 10 min. The supernatant was collected by centrifugation at 12,000 × *g* for 20 min at 4 °C. The protein concentration was determined using the BCA Protein Assay Kit to ensure consistency across groups, followed by boiling at 99 °C for 10 min prior to loading. After transfer at room temperature, the membrane was blocked with 5% skimmed milk in TBST for 2 h. Subsequently, the membrane was incubated with the primary antibody at 4 °C overnight, followed by incubation with the secondary antibody at room temperature for 1 h. The membrane was then developed using ECL solution, and quantitative analysis was performed using ImageJ. All related experimental reagents, including NOTCH1, DDIT3, XBP1s, phospho-EIF2α, ATF6, caspase-3, and caspase-7 were purchased from Beijing Biosynthesis Biotechnology Co., Ltd.

### Cardiomyocyte culture

Under the microscope, dissect the hearts from E14.5 mouse embryos and place them in a pre-cooled PBS solution at 4 °C. Wash the hearts with PBS, retaining only the apices. Mince the tissue, add trypsin, and digest in a 37 °C water bath for 10 min. Remove the supernatant, add trypsin again, and digest at 37 °C for another 10 min, retaining the supernatant. Repeat this process 5–8 times until the tissue fragments become transparent. Pass the cell suspension through a 200-mesh sieve, centrifuge at 1000 rpm for 5 min, remove the supernatant, resuspend in complete DMEM culture medium, and transfer to a culture dish for differential adhesion culture. After 1.5 h of culture, aspirate the non-adherent cells from the dish and transfer them to another dish, then place in a 37 °C, 5% CO2 incubator for culture until the cells extend pseudopods and begin synchronous pulsation. To assess the effect of the IRE1α pathway on NOTCH1 expression in cardiomyocytes, IRE1α inhibitor 4-methyl umbelliferone 8-carbaldehyde (4μ8c) (16) was added to the A, B, and C groups, with an equivalent dose of DMSO solution used as a control. After co-culture, the cells were collected, and the expression of relevant proteins was analyzed. Detailed methods can be found in (17).

### Quantitative real-time PCR analysis

Total RNA was extracted from tissue specimens using the TRIzol reagent, and RNA integrity and purity were assessed with a nucleic acid spectrophotometer. First-strand complementary DNA (cDNA) was synthesized from 1 μg of total RNA using a reverse transcription kit [Yeasen Biotechnology (Shanghai) Co., Ltd.] according to the manufacturer’s instructions. Quantitative real-time PCR was performed on a real-time PCR detection system with SYBR Green chemistry under standard cycling conditions. GAPDH was used as the endogenous control, and relative mRNA expression levels were calculated using the comparative Ct method (2^∧^-ΔΔCt). The primer sequence used for quantitative real-time PCR was as follows: NOTCH1:

Forward: 5′-TGGAGACAGGCAACAGTGAGGAA-3′,
Reverse: 5′-CTTGGCAGCATCTGAACGAGAGTAT-3′.

### Statistical analysis

All statistical analyses were conducted using GraphPad Prism 9. The serological structures and protein staining intensities of all control and experimental groups were compared using one-way ANOVA. The data presented in the figures are expressed as mean ± sd.

## Results

(1) Successfully established a HFD model

**FIGURE 1:**
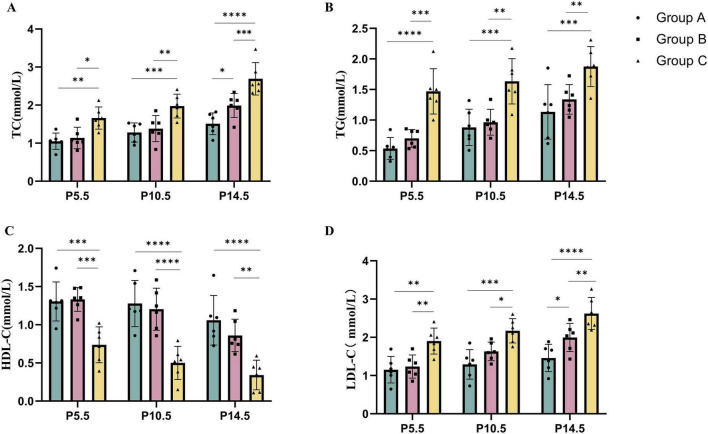
High-fat diet (HFD) during pregnancy or HFD before and during pregnancy leads to dyslipidemia in maternal mice. The levels of TC **(A)**, TG **(B)**, HDL-C **(C)**, and LDL-C **(D)** in the serum of pregnant mice (*n = 6*) receiving different diets before and during pregnancy were measured at the time points of pregnant day 5.5 (P5.5), P10.5, and P14.5. Data were displayed as mean ± sd. *p < 0.05, **p < 0.01, ***p < 0.001, ****p < 0.0001. TG, triglycerides; TC, total cholesterol; HDL-C, high-density lipoprotein cholesterol; LDL-C, low-density lipoprotein cholesterol; HFD, high-fat diet.

To investigate the effects of a high-fat diet (HFD) administered before, and during pregnancy on maternal serum lipid profiles, we measured total cholesterol (TC), triglycerides (TG), low-density lipoprotein cholesterol (LDL-C), and high-density lipoprotein cholesterol (HDL-C) levels in maternal blood at gestational days 5.5, 10.5, and 14.5. As shown in Figure 1, at P5.5, maternal mice in the C group exhibited higher levels of TC, TG, and LDL-C, but lower HDL-C levels compared with those in the A group. This trend became more pronounced with the prolonged duration of HFD exposure. Furthermore, by P14.5, TC and LDL-C levels in the B group were higher than those in the A group. These results suggest that maternal dyslipidemia is positively correlated with the duration of HFD intake–the longer the exposure, the earlier and more severe the onset of lipid abnormalities. In summary, a maternal HFD mouse model was successfully established in this study.

(2) Maternal HFD during pregnancy leads to abnormal cardiac structure in offspring

**FIGURE 2:**
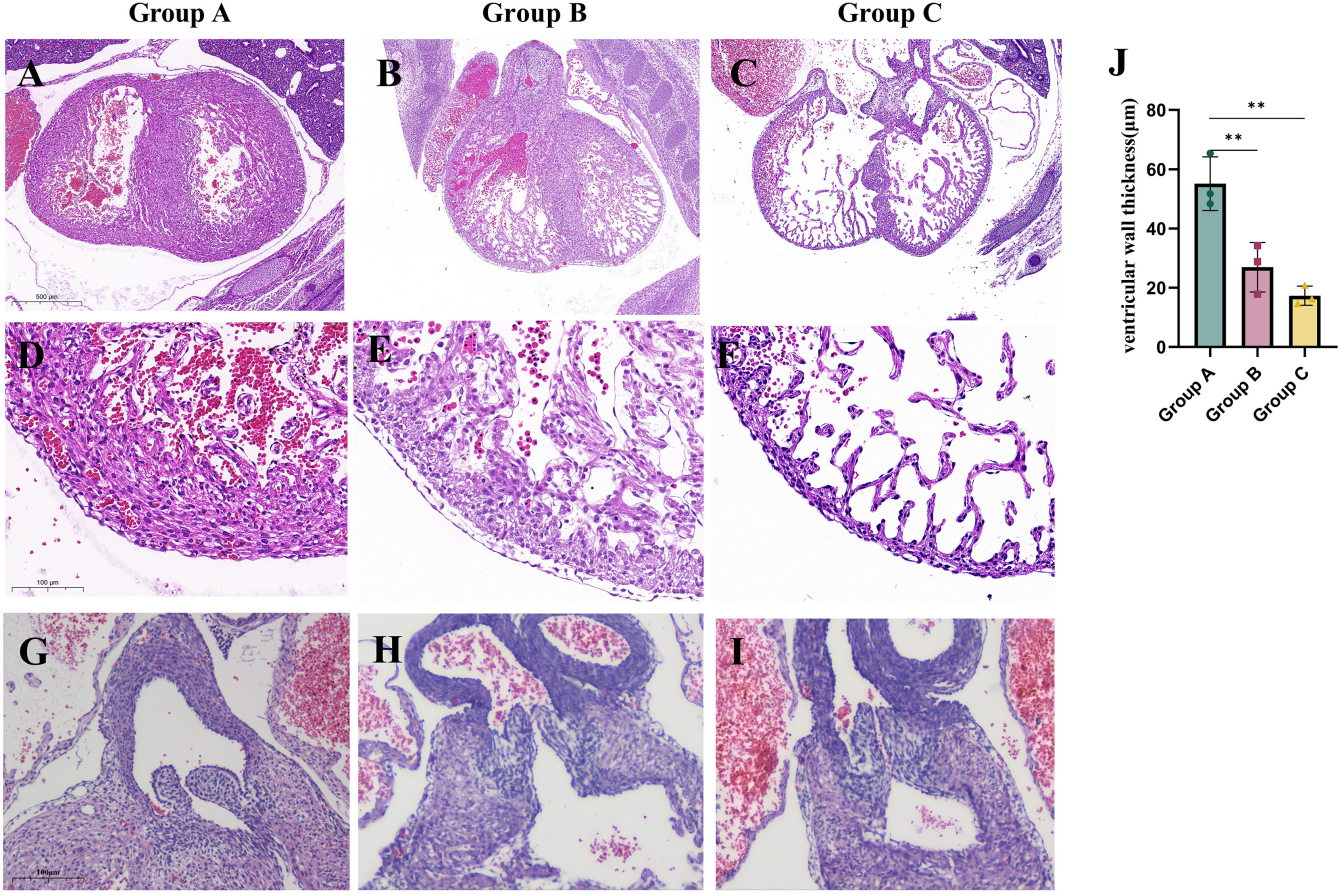
Maternal consumption of a HFD during pregnancy leads to abnormal cardiac structure in embryonic mice. The morphology of E14.5 embryonic mouse hearts in each study group. **(A–C)** The morphology of the ventricles in embryonic mice from each study group. (**D—F,J**) Comparison of ventricular wall thickness in embryonic mice from each study group (*n = 3*). **(G–I)** The morphology of the valves in embryonic mice from each study group. Data were displayed as mean ± sd. **p < 0.01.

To evaluate the impact of a maternal HFD on cardiac development in offspring, we examined the cardiac tissue structure of embryonic mice at E14.5. Our findings indicate that maternal consumption of a HFD during pregnancy leads to abnormal cardiac structures in the offspring. In the A group, dense arrangements of cardiomyocytes were observed forming the ventricular walls and compact cardiac trabeculae. In contrast, the B group exhibited loose cardiomyocyte arrangements, significantly thinner ventricular walls, and reduced density of ventricular trabeculae. Furthermore, in C group, the ventricular walls were even thinner, and the trabeculae were more sparse (Figures 2A–F). Additionally, maternal HFD also influenced the development of valves in the embryonic hearts. In the A group, the embryonic heart valves were slender, while those in the B and C groups were dysplastic (Figures 2G–I). These findings suggest that a HFD during pregnancy negatively affects embryonic heart development, with an increased duration of maternal HFD correlating with more severe structural malformations in the offspring’s heart.

(3) Maternal HFD during pregnancy leads to decreased expression of NOTCH1 protein in embryonic hearts

**FIGURE 3:**
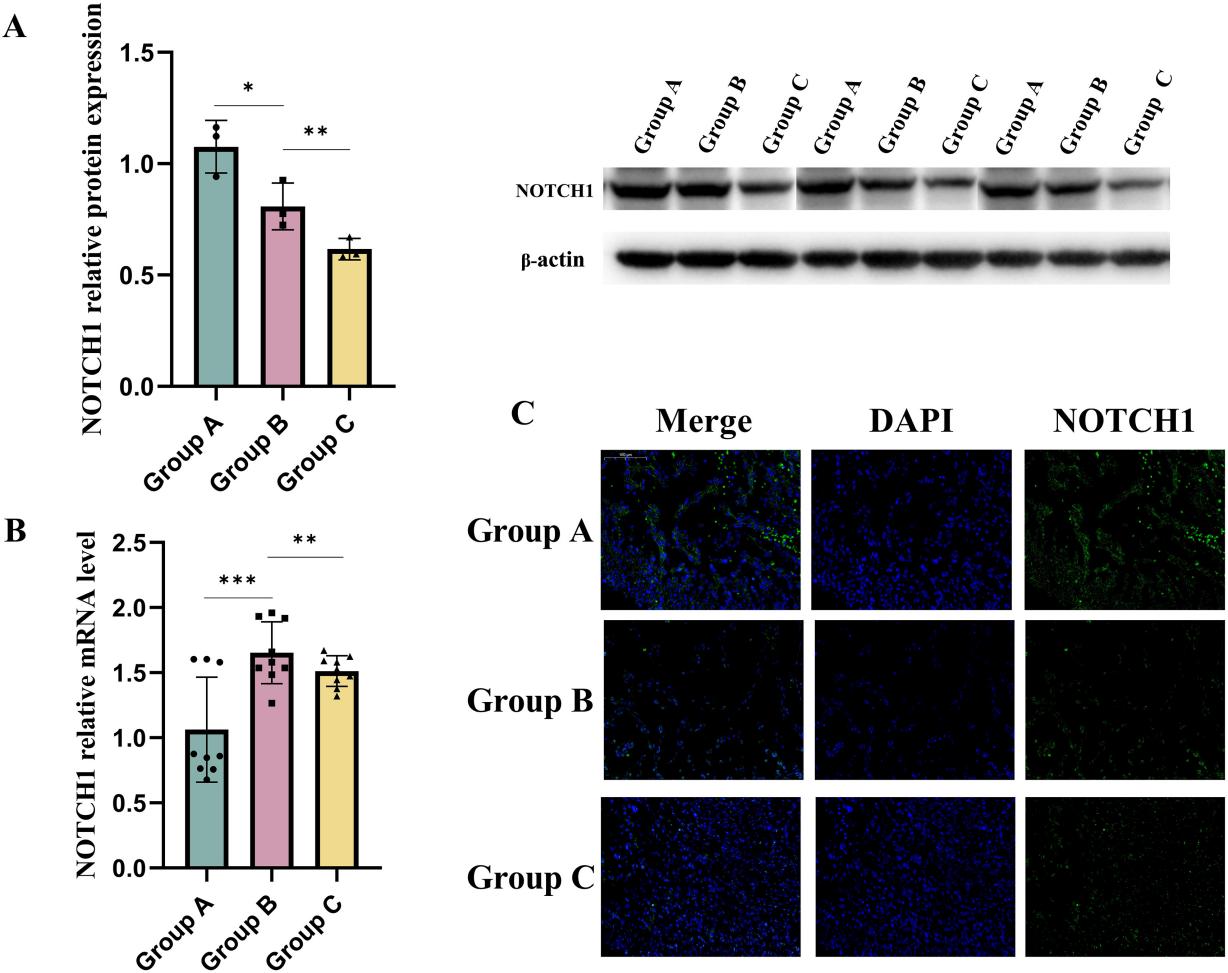
Maternal high-fat diet during pregnancy leads to a decrease in NOCTH1 expression in the embryonic mouse ventricle. The transcriptional **(B)** and translational **(A,C)** levels of NOTCH1 in the embryonic mouse ventricles of each study group. Data were displayed as mean ± sd. *p < 0.05, **p < 0.01, ***p < 0.001.

NOTCH1 plays a crucial role in the differentiation and proliferation of human ventricular-like cardiomyocytes (18). The receptors and ligands associated with NOTCH signaling are predominantly expressed in the developing endocardium and myocardium, facilitating the differentiation of endothelial cells into ventricular trabeculae (19). Concurrently, NOTCH1 activates BMP10 in cardiomyocytes, which promotes cardiomyocyte proliferation (20). During the development of the mouse ventricular chamber, NOTCH signaling initially connects the endocardial layer with cardiomyocytes to support ventricular trabeculation. Subsequently, it coordinates ventricular morphogenesis and compaction alongside coronary artery development, ultimately leading to the formation of a mature ventricle (21). Therefore, we hypothesize that the impaired trabeculation and hindered compaction of the ventricular wall in the offspring of dams fed a HFD result from the inhibition of NOTCH1 signaling in the hearts of these offspring. We evaluated the expression of NOTCH1 in the embryonic hearts of different experimental groups using Western blot analysis and Immunofluorescence staining found that maternal consumption of a HFD significantly reduces NOTCH1 signaling in the embryonic hearts (Figures 3A, C). QPCR analysis of NOTCH1 revealed that, compared with group A, transcriptional levels of NOTCH1 were elevated in the hearts of embryos from groups B and C. This increase is likely a compensatory response to translational inhibition of NOTCH1 in these groups (Figure 3B).

(4) Maternal HFD can induce unfolded protein response in offspring hearts, leading to translational repression of NOCTH1

**FIGURE 4:**
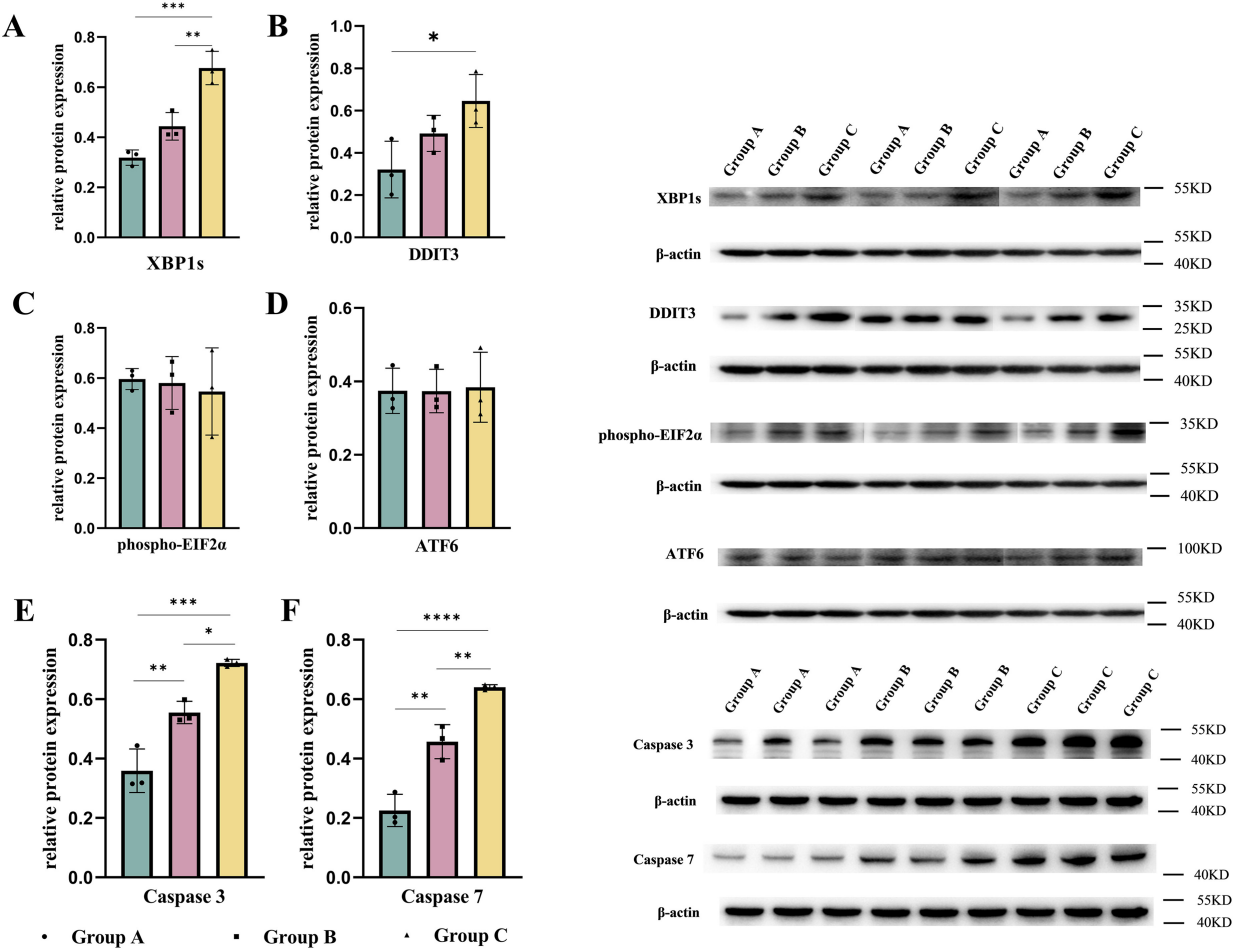
Maternal high-fat diet during pregnancy activates the unfolded protein response and induces apoptosis in the ventricles. The expression of UPR-related signaling molecules **(A–D)** and caspase-3, 7 **(E,F)** in the embryonic mouse ventricles of each study group. Data were displayed as mean ± sd. *p < 0.05, **p < 0.01, ***p < 0.001, ****p < 0.0001.

**FIGURE 5:**
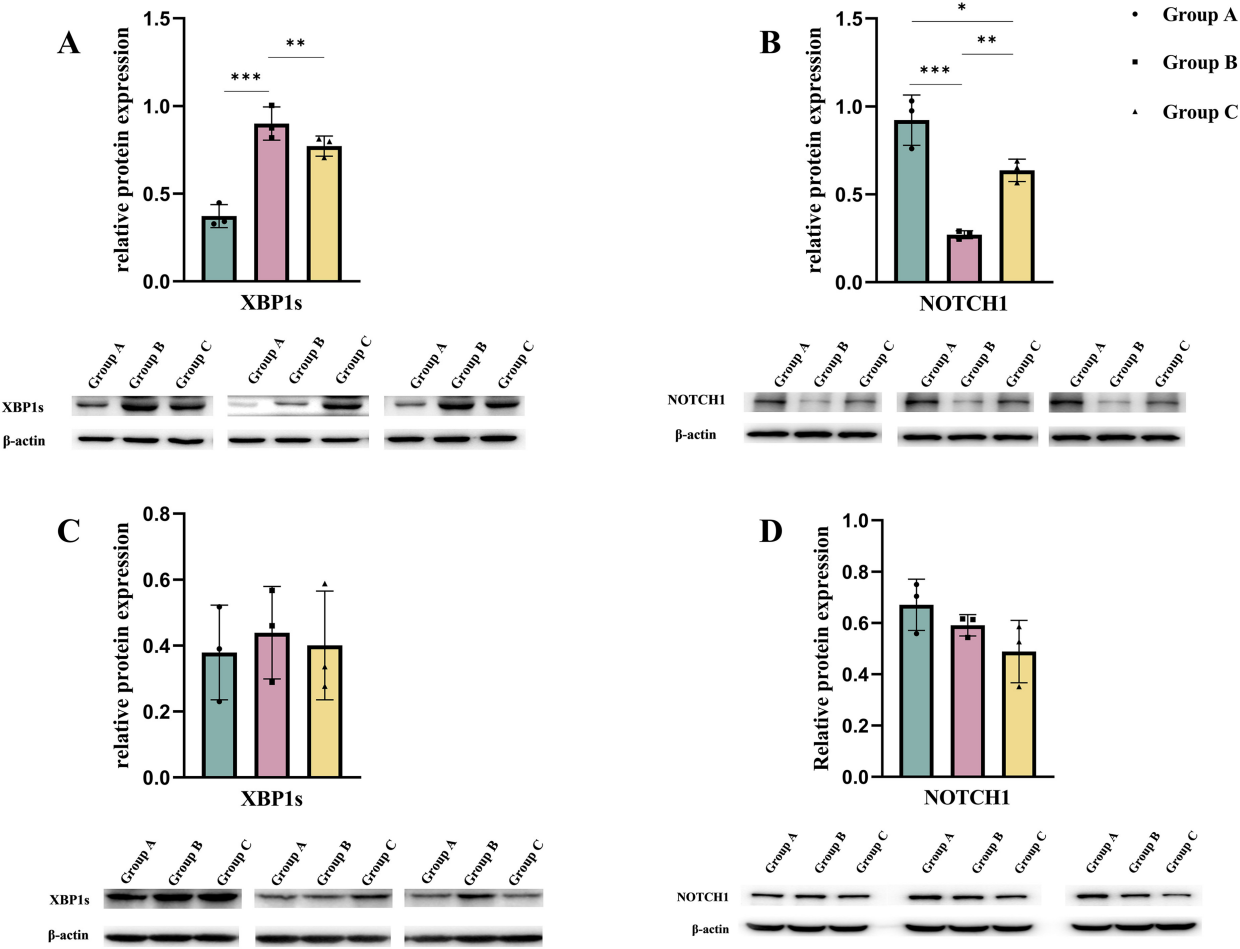
By culturing embryonic mouse cardiomyocytes from each study group *in vitro*, the expression of NOTCH1 in the cells was observed after blocking the IRE1α pathway. **(A,B)** Expression of XBP1s and NOTCH1 in embryonic mouse cardiomyocytes of each study group when the IRE1α pathway was not blocked. **(C,D)** Expression of XBP1s and NOTCH1 in each study group after co-culturing with the IRE1α inhibitor 4μ8c added to the cell culture medium. Data were displayed as mean ± sd. *p < 0.05, **p < 0.01, ***p < 0.001.

Maternal consumption of a HFD leads to lipid accumulation in the cardiomyocytes of offspring, inducing ER stress. To mitigate this stress and restore ER homeostasis, the UPR is activated. However, prolonged activation of the UPR can result in cellular dysfunction and even cell death (22, 23). We measured the UPR marker DNA Damage-Inducible Transcript 3 (DDIT3) (24) and found that DDIT3 levels were significantly elevated in both the B and C groups. Consistent with this result, the levels of key apoptosis-related proteins, Caspase-3 and Caspase-7 (25), were significantly increased in the hearts of embryos from groups B and C. Additionally, we examined the expression of UPR-related signaling pathways in embryonic mouse heart tissues. Among these pathways, the downstream effector of IRE1α, XBP1s, was significantly increased in both the B and C groups, while no significant differences were observed in the downstream molecules of the PERK pathway, phospho-EIF2α, and the ATF6 pathway. This indicates that maternal consumption of a HFD activates the UPR in the endoplasmic reticulum of the embryonic heart, primarily through the IRE1α pathway (Figure 4). To further validate the translational inhibitory effect of the IRE1α pathway on NOTCH1 signaling, we cultured E14.5 embryonic mouse cardiomyocytes *in vitro* and added the IRE1α pathway inhibitor 4μ8c to the cardiomyocytes in each study group. We found that after blocking the IRE1α pathway, the inhibition of NOTCH1 expression in the B and C groups improved compared to before, and there was no significant difference when compared to the A group (Figure 5). This suggests that a maternal HFD during pregnancy may inhibit the translation of NOTCH1 by activating the IRE1α pathway in the embryonic mouse hearts of the offspring.

## Discussion

High-fat diet are increasingly prevalent in dietary patterns across various countries, contributing to rising global obesity rates, particularly among women of childbearing age and pregnant women (24, 26). Obese pregnant women are at a heightened risk of hypertensive disorders and gestational diabetes, alongside a greater incidence of adverse pregnancy outcomes such as stillbirth, preterm birth, macrosomia, and congenital malformations (5, 27). Moreover, elevated maternal lipid levels can adversely affect the long-term health of the child. Elevated maternal TC and LDL-C levels enhance placental transfer of fetal TC and LDL-C, potentially leading to the formation of fatty streaks in fetal blood vessels, thereby increasing the offspring’s risk of cardiovascular issues (28). Additionally, high intrauterine concentrations of TC or TG may impair pancreatic beta cell function in the offspring, thereby increasing the likelihood of early islet dysfunction, insulin resistance, and cardiac-related diseases in later life (29). From P0.5 to P14.5, maternal levels of TC, TG, and LDL-C in the HFD pregnant group were significantly higher than those in the normal-fat diet pregnant group, while HDL-C levels were significantly lower in the C group compared to the A group. This indicates that long-term maternal HFD consumption leads to maternal dyslipidemia during pregnancy, which subsequently results in abnormal cardiac structure in the embryo. Interestingly, at P5.5 and P10.5, the B group exhibited non-significant differences in TC, and LDL-C compared to the A group, with significant differences emerging only at P14.5. The embryonic heart development in the B group was still affected, underscoring the importance of maintaining a healthy diet during pregnancy. Even short-term dietary changes can significantly impact embryonic development.

An increasing body of evidence suggests that maternal obesity can have lifelong negative effects on the cardiac health of offspring (30). Studies have demonstrated that feeding mother rats a HFD during pregnancy increases the risk of cardiovascular disease in their offspring by impairing mitochondrial function in the offspring’s hearts (31) and enhancing sympathetic nervous system activity (32). Furthermore, a HFD during pregnancy can influence the development of the embryonic heart through epigenetic modifications, resulting in structural abnormalities. Specifically, when mother rats were fed a high-palmitic acid diet during pregnancy, the nuclear factor κB p65 pathway in the embryonic heart was activated, leading to the homocysteinylation of lysine in the GATA4 protein, which inactivates GATA4 and contributes to atrial septal defects or ventricular septal defects in the embryos (33). In this study, at E14.5, embryonic hearts in the B and C groups exhibited thinning of the ventricular wall, enlargement of the trabeculated region, and impaired valve development, consistent with the manifestations of disrupted NOTCH1 signaling. NOTCH1 plays a crucial role in the fate determination and morphogenesis of cardiac cells during human heart development. In the ventricular chamber development process, NOTCH1 initially connects the endocardial layer and the myocardial layer to support ventricular trabeculation, and subsequently coordinates with coronary development to shape the ventricular crest and compact the ventricular wall, ultimately forming mature ventricles (34). Therefore, we examined NOTCH1 expression in the embryonic mouse hearts across the study groups, finding that NOTCH1 protein levels were significantly lower in the B and C groups compared to the A group.

A significant number of initially secreted proteins require proper folding within the ER, which imposes continuous stress on the ER. Under conditions of ER stress, cells activate the UPR to mitigate fluctuations in the load of unfolded proteins (35). When cells experience irreversible stress, the UPR facilitates the elimination of damaged cells through apoptosis (36). Adverse maternal environments can act as stressors, inducing the UPR in embryonic mouse hearts, including hypoxia (16), viral infections, and hyperthermia (37). One identified cause of adverse intrauterine environments is a HFD during pregnancy, which increases oxidative stress in the offspring’s heart and elevates cardiomyocyte apoptosis (11). Our experiment demonstrated that a HFD during pregnancy activates the UPR via the IRE1α pathway. In the presence of ER stress, the IRE1α protein enhances the downstream active transcription factor XBP1s. XBP1s can upregulate the transcription of KLF9 by binding to the unfolded protein response element on its promoter. KLF9 further promotes the release of Ca^2 +^ from the ER by increasing the transcription of ER calcium storage regulatory protein 38B and inositol 1,4,5-trisphosphate receptor type 1, ultimately leading to cell death (38). When we treated the cardiomyocyte culture medium of embryonic mice in each research group with the small molecule inhibitor of IRE1α, 4μ8c, the differences in NOCTH1 expression among the groups were eliminated.

Abnormalities in embryonic heart structure are associated with HFD intake during pregnancy, underscoring the significance of the intrauterine environment provided by the mother for embryonic development. Notably, in this experiment, the degree of cardiac abnormalities in the C group of embryonic mice was greater than that in the B group, suggesting that mechanisms beyond the UPR are also involved. Possible explanations include the alteration of the maternal metabolic environment due to a HFD prior to conception, the induction of a pro-inflammatory fat profile in the mother, modifications to uterine architecture and ovarian follicle profiles (39), as well as abnormalities in mitochondrial ultrastructure and size in oocytes (40). Given that heart development necessitates precise spatiotemporal expression of relevant genes, maternal consumption of a HFD, whether before or during pregnancy, can disrupt normal embryonic heart development. The IRE1α pathway within the UPR signaling cascade represents just one of the mechanisms implicated. Any disruption in genetic development during embryonic heart formation can result in abnormal heart development. Therefore, avoiding a HFD before and during pregnancy is the optimal strategy to prevent abnormal embryonic heart development.

Our research indicating that a maternal HFD can adversely affect the normal development of the embryonic heart. However, our study also has certain limitations. The mechanism by which the IRE1α pathway mediates changes in NOTCH1 expression in the embryonic mouse heart remains unclear, and this is an area that we will explore in our subsequent research. Additionally, the impact of a maternal pre-pregnancy HFD on embryonic heart development requires further investigation. In summary, this study demonstrates that maternal consumption of a HFD before and during pregnancy activates the UPR and IRE1α signaling in the embryonic heart, leading to a reduction in Notch1 expression, impaired compaction of the ventricular wall in the offspring, thinning of the ventricular wall, and abnormal development of the heart valves. Therefore, the intake of a normal diet before and during pregnancy is critical for the normal development of the embryonic heart.

## Data Availability

The original contributions presented in this study are included in this article/Supplementary material, further inquiries can be directed to the corresponding author.
