# Supplementary material for: NOTCH1 and UPR signaling in embryonic heart development under maternal high-fat diet influence
Source: Front Med (Lausanne). 2025 Dec 4;10:1620495. doi: 10.3389/fmed.2025.1620495 (PMC12711718; doi:10.3389/fmed.2025.1620495)
Supplement: Supplementary file 1 [file Table_1.doc]

**Table S1. Ingredient composition of control (D12450B) and high-fat (D12492) diet for mice.**

| **Ingredient** | **D12450B (g)** | **D12492 (g)** |
| --- | --- | --- |
| Casein | 200 | 200 |
| L-Cystine | 3 | 3 |
| Corn Starch | 351 | 0 |
| Maltodextrin | 35 | 125 |
| Sucrose | 350 | 68.8 |
| Cellulose | 50 | 50 |
| Soybean Oil | 25 | 25 |
| Lard | 20 | 245 |
| Mineral Mix | 10 | 10 |
| DiCalcium Phosphate | 13 | 13 |
| Calcium Carbonate | 5.5 | 5.5 |
| Potassium Citrate, 1 H2O | 16.5 | 16.5 |
| Vitamin Mix V10001 | 10 | 10 |
| Choline Bitartrate | 2 | 2 |
| **Total** | 1055.05 | 773.85 |

Mass indicates the amount of each ingredient used to prepare the diet (g).
